# The Effect of Physical Activity/Exercise on miRNA Expression and Function in Non-Communicable Diseases—A Systematic Review

**DOI:** 10.3390/ijms25136813

**Published:** 2024-06-21

**Authors:** Moomna Afzal, Francesca Greco, Federico Quinzi, Francesca Scionti, Samantha Maurotti, Tiziana Montalcini, Annamaria Mancini, Pasqualina Buono, Gian Pietro Emerenziani

**Affiliations:** 1 Department of Clinical and Experimental Medicine, University Magna Grecia, 88100 Catanzaro, Italy; moomna.afzal@studenti.unicz.it (M.A.); fquinzi@unicz.it (F.Q.); scionti@unicz.it (F.S.); smaurotti@unicz.it (S.M.); tmontalcini@unicz.it (T.M.); 2 Department of Movement, Human and Health Sciences, Foro Italico University of Rome, 00135 Rome, Italy; f.greco2@studenti.uniroma4.it; 3 Research Center for the Prevention and Treatment of Metabolic Diseases, University Magna Grecia, 88100 Catanzaro, Italy; 4 Department of Medicine, Movement Sciences and Wellbeing, University Parthenope, 80133 Naples, Italy; annamaria.mancini@uniparthenope.it (A.M.); buono@uniparthenope.it (P.B.); 5 CEINGE-Biotecnologie Avanzate Franco Salvatore s.r.l, 80131 Naples, Italy

**Keywords:** micro-RNAs, c-miRNAs, myo-miRNAs, cancer, cardiovascular diseases, chronic obstructive pulmonary disease (COPD), type 2 diabetes mellitus (T2DM)

## Abstract

Exercise may differently affect the expression of key molecular markers, including skeletal muscle and circulating miRNAs, involved in cellular and metabolic pathways’ regulation in healthy individuals and in patients suffering from non-communicable diseases (NCDs). Epigenetic factors are emerging as potential therapeutic biomarkers in the prognosis and treatment of NCDs and important epigenetic factors, miRNAs, play a crucial role in cellular pathways. This systematic review aims to underline the potential link between changes in miRNA expression after different types of physical activity/exercise in some populations affected by NCDs. In June 2023, we systematically investigated the following databases: PubMed, MEDLINE, Scopus, and Web of Science, on the basis of our previously established research questions and following the PRISMA guidelines. The risk of bias and quality assessment were, respectively, covered by ROB2 and the Newcastle Ottawa scale. Of the 1047 records extracted from the initial search, only 29 studies were found to be eligible. In these studies, the authors discuss the association between exercise-modulated miRNAs and NCDs. The NCDs included in the review are cancer, cardiovascular diseases (CVDs), chronic obstructive pulmonary disease (COPD), and type 2 diabetes mellitus (T2DM). We evidenced that miR-146, miR-181, miR-133, miR-21, and miRNA-1 are the most reported miRNAs that are modulated by exercise. Their expression is associated with an improvement in health markers and they may be a potential target in terms of the development of future therapeutic tools.

## 1. Introduction

The human genome contains more than 2500 mature miRNAs that control gene expression in a wide range of physiological and pathological cellular processes [1]. Most of the miRNA are present within the cells, but others, known as circulating miRNAs (c-miRNAs), have been identified in extracellular body fluids (e.g., plasma, serum, saliva, and urine). Since the c-miRNAs’ function is to act as intercellular signaling molecules establishing cell–cell communication [2], these could also be considered as potential non-invasive biomarkers for multiple non-communicable diseases (NCDs) [1].

The difference in the expression patterns of miRNAs and c miRNAs is linked to the most frequently observed epigenetic dysregulations resulting from NCDs [3,4], such as inflammation, cardiovascular disease, obesity, muscle hypertrophy, lymphomas, leukemia, and cancer [5,6].

Furthermore, growing research also supports the theory that physiological conditions (e.g., age and pregnancy), environmental factors (e.g., drugs, radiation, and viruses), and lifestyle choices (e.g., exercise, stress, nutrition, diet, alcohol, and cigarettes) all have an impact on miRNA expression [7,8,9]. More specifically, it can be said that exercise regulates the expression of many miRNAs that, in turn, control the expression patterns of several genes. Similarly, much evidence has been provided regarding the effects of different types of exercise (e.g., aerobic or resistance) and times of exertion (acute or chronic) on the expression of miRNAs in different organs [10,11], leading to increased protein synthesis [12].

It is known that physical activity/exercise promotes health by inducing a physiological improvement in different tissues and organs in healthy individuals and in those with non-communicable diseases (NCDs) [13]. Indeed, observational and randomized trials have shown that regular exercise contributes to preventing and managing most of the common NCDs. Considerable evidence concerns the role of exercise in the primary and secondary prevention in cardiovascular diseases (CVD), hypertension, as well as metabolic disorders (type 2 diabetes mellitus, obesity, and dyslipidemia) or in muscle, bone, and joint diseases (chronic fatigue syndrome and osteoporosis). However, only limited evidence has been provided so far on the effects mediated by exercise on chronic obstructive pulmonary disease (COPD) and in some types of cancers [14,15]. 

Moreover, during exercise-induced physiological improvements, the expression of different miRNAs, including myo-miRNAs and/or c-miRNAs, appears to be affected. Therefore, the investigation into exercise-induced miRNA expression modifications in NCDs may open up new perspectives on the epigenetic adaptations induced by exercise. However, despite the potential role of miRNAs in mediating health-related improvements, the molecular mechanisms are still poorly understood among individuals affected by NCDs [16]. 

Previous studies focused on the identification of myo-c-miRNAs differently expressed in association with acute and chronic exercise in humans [17,18]. Moreover, these investigations primarily focused on improving health outcomes within different patients, such as those affected by cancer or cardiovascular disorders (CVD) [19,20]. 

The aim of this systematic review is, therefore, to summarize the state-of-the-art regarding the effects mediated by different types of exercise on the expression of myo- or c-miRNAs in various NCDs, in association with the improvement of health outcomes, and to speculate on the molecular pathways involved in this process.

## 2. Results 

### 2.1. Research Selection 

**Figure 1:**
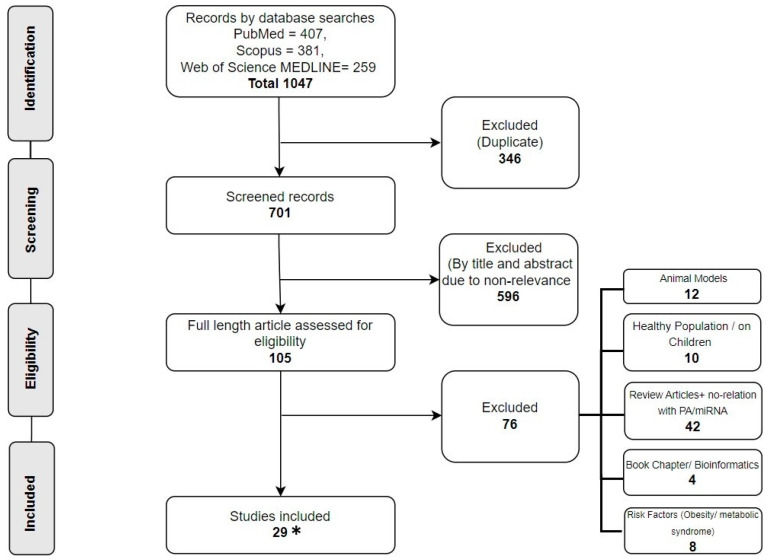
The PRISMA flow diagram of the study selection process. * the final actual number of included studies are 28.

A total of 1047 articles were retrieved after the search strategy was implemented. After removing the duplicates, 701 records were assessed for eligibility criteria. At this stage, each author (M.A. and F.G.) worked independently on the spreadsheet, and disagreements were settled through discussion. Following this screening, 596 records were deleted on the basis of non-relevance of the title and abstract, leaving 105 manuscripts for the second phase of the second screening. Indeed, 76 manuscripts were excluded due to their focus on animal models, healthy population, review articles, book chapters, risk factors (i.e., obesity and metabolic syndrome), and lack of relation with PA and miRNA. Therefore, 29 studies were included for systematic review. However, as an included study was retrieved afterwards, the final included studies are 28. The flowchart presented in Figure 1 provides a detailed description of the research selection process.

### 2.2. Study Characteristics 

Five articles regarding cancers, thirteen on cardiovascular diseases (CVDs), nine on type 2 diabetes mellitus (T2DM), and two on chronic obstructive pulmonary disease (COPD) were retrieved.

All the included studies focused on the diseased human population. Furthermore, most of these studies investigated circulating miRNAs in the plasma and serum, while only five studies addressed the regulation of miRNAs in tissue biopsies.

### 2.3. Cancer

One of the studies focused on the relationship between physical activity/exercise and miRNA expression in prostate cancer, and four studies focused on patients with breast cancer. With regard to the studies on cancer, a total of 225 individuals, including 23 males with prostate cancer (PC) and 202 females with breast cancer (BC), were investigated. The mean age of participants varied between 30 and 78 years old. The duration of the exercise interventions varied between 12 and 24 weeks.

Gazova analyzed the effects of 16 weeks of strength training (ST) in a sample of 23 prostate cancer (PC) males with androgen-deprivation therapy (ADT) for the expression of a tumor suppressor miRNA, miR-133a [21]. Results showed that miR-133a was upregulated in the ST group with ADT [22]. Moreover, the upregulation of miR-133a-3pa also emerged in female breast cancer (BC) survivors after 16 weeks of resistance training on leg and chest press for 60 min, 3 times a week [23].

Exercise training in both PC and BC survivors showed upregulation of the circulating microRNA-1, miR-29b, and miR-370-3p, a marker of tumor suppression associated with myogenesis [21,23]. It has been shown that a period of 12 weeks of moderate physical exercise or strength training or high-intensity interval training (HIIT) increases the expression of tumor suppressor miRNAs, miR-211, miR-205, miR-10a, and miR-206, in early-stage BC patients undergoing hormone therapy (HT) [22,24]. Two other studies investigated the expression of tumor suppressors and oncomiRs in BC patients undergoing hormone therapy [24,25]. Both studies showed downregulation of an oncomiR, miR-27a, after exercise [24,25]. Moreover, Alizadeh reported increased expression of tumor suppressor miRNAs, i.e., miR-206, miR-145, miR-143, miR-9, and let-7a, and decreased expression of oncomiRs, i.e., miR-21, miR-155, miR-221, and miR-10b, after high-intensity interval training (HIIT) for 12 weeks [24]. Adams found downregulation of miR-106b and miR-92a after six months of aerobic exercise (20 min/day) and weight loss, in correlation with body mass index (BMI), in post-menopausal BC survivors treated with hormones and exercise (HOPE) [25]. The miRNAs that emerged are potential prognostic markers of BC recurrence. Moreover, a negative correlation has been reported between the four RNAs (miR-191, miR-17, miR-103a, and miR-93) and BMI [25].

### 2.4. Type 2 Diabetes Mellitus (T2DM)

A total of 169 individuals with type 2 diabetes mellitus (T2DM) were investigated across all the included studies. The mean age of participants varied from 45 to 68 years of age. The duration of the exercise intervention varied between acute exercise (40 min) and chronic exercise (24 weeks).

Akbarinia analyzed the effects of eight weeks of aerobic training (AT; 60–75% VO_2peak_) on miR-130a expression in a sample of females affected by T2DM. Although it was mostly upregulated in the AT group, no significant differences in miR-130a expression between the trained and untrained groups emerged [26]. Moreover, a prospective observational study found that miR-130a expression was upregulated in the moderate exercise group (500 MET/week) monitored for 12 weeks [27]. The same study illustrated that in the high-intensity trained group (from 750 to 999 MET-minutes/week), miRNA-146a was downregulated. MicroRNA-146a is a marker of a senescence-associated pro-inflammatory status in vascular cells [27]. Two other studies investigating miR-146a expression revealed an upregulation after resistance training (RT) [28] and after eight weeks of a combination of RT (40–70% of 1-RM) and HIIT [29] in diabetic patients. Moreover, these positive effects occurred with different modes of exercise administration (same day vs. different days) [28,29]. It must also be mentioned that miR-29b was downregulated only following combined RT and HIIT performed on separate days [29].

Exercise training in T2DM is associated with substantial c-miRNA profile changes, irrespective of exercise type [30]. Indeed, levels of miR-423-3p, miR-451a, and miR-766-3p were upregulated after 24 weeks of both AT (60–65% of HRR) and RT (70–80% 1-RM) [30].

Regulation of the transcriptome downstream of miR-29a suggests a novel epigenetic mechanism controlling therapeutically functional vascular plasticity in skeletal muscle in aerobic training, which is worthy of further investigation associated with therapeutic interventions for vascular disease. Meanwhile, the response to RT upregulation of miR-23a and miR-195 had an inverse-expression-binding association with genes involved in blood vessel development [31].

Furthermore, Simaitis evidenced that miRNA-29b-3p, miRNA-29c-3p, and miRNA-135a-5p expression were downregulated in the skeletal muscle of T2DM patients after 12 weeks of AT (70–80% of peak heart rate) [32].

Although it has been reported that the attenuated expression of hsa-miR-223 in plasma and platelets is a marker of thrombotic events in individuals affected by T2DM, no effects of AT (60–75% of VO_2peak_) on hsa-miR-223 expression were found after eight weeks of training compared to a non-exercise group [33]. On the contrary, an upregulation of miRNA-223 after 12 weeks of AT (65–75% of VO_2peak_) was reported [34].

### 2.5. Chronic Obstructive Pulmonary Disease (COPD)

A total of 35 individuals with COPD were investigated across all the included studies. The mean age of participants varied from 60 to 67 years of age. The duration of the exercise intervention varied from one day to twelve weeks.

In an observational study, miR-133 and miR-206 were negatively correlated with daily PA [35]. A pilot study revealed that miR-144-3p and hsa-miR-1277 were downregulated after 12 weeks of an individualized AT, whereas hsa-let-7c was upregulated [36].

### 2.6. Cardiovascular Diseases (CVDs)

A total of 341 individuals with cardiovascular disease (CVD) were investigated across all the included studies. The mean age of participants varied from 35 to 70 years of age. The duration of the exercise intervention varied from acute (15 min) to chronic (16 weeks).

Antunes analyzed the effects of 16 weeks of aerobic exercise (AE; 60–72% VO_2peak_) in a sample of 34 adults affected by heart failure with reduced ejection fraction (HFpEF) on miRNA-146 expression, an inflammation marker. After exercise training, a decrease in muscle inflammation was observed, as indicated by increased miRNA-146 levels and the stable NF-κB/IκB-α ratio in muscle tissues [37]. However, two studies reported the upregulation of miR-146a [10] in exercise non-responders (ENR) and miR-146a-5p [38] in 24 coronary artery disease (CAD) patients. In the study by Witvrouwen, patients underwent 16 weeks of AE. They were HFrEF-suffering males with 90% of heart rate (HR) [10]. In another study by Witvrouwen, the patients underwent 15 weeks of aerobic interval training (AIT) compared to moderate continuous training (MCT), respectively [39]. Moreover, Witvrouwen analyzed the effects of 15 weeks of combined strength and AT (at 90% of HR) in adults affected by HFpEF on miRNA-146 expression, which was downregulated after the training protocol, compared to healthy matched individuals [39].

According to Antunes-Correa, aerobic training (AT), but not inspiratory muscle training (IMT), was able to upregulate microRNA-1 in HFpEF patients (oxygen uptake ≤ 20), consequently reducing the PI3K-AKT pathway and increasing the functional capacity of muscles and blood flow in legs [40]. However, Barbara Mayr found no change in miRNA-1 nor in miR-133, miR-208a, and miR-499 after maximum-cycle ergospirometry in 20 CAD patients [41]. To check miRNA-181c in HFpEF patients (70 ± 6 years), Gevaert enrolled 51 patients in AT (peakVO2 > 6.4%), completing moderate continuous training (MCT) and high-intensity interval training (HIIT) for 12 weeks [42]. The results demonstrated downregulation of miRNA-181c in individuals with low compliance to the exercise compared to those with high compliance [42]. Witvrouwen identified a set of seven upregulated miRs, i.e., Let-7b, miR-23a, miR-140, miR-146a, miR-191, miR-210, and miR-339-5p, in ENR after four months of aerobic exercises in HFrEF patients (50.7–65.4 years) and in nine exercise non-responders (ENR; VO_2peak_ of <6%). These miRNAs were highly correlated with VO_2peak_ trainability in HFrEF patients [10]. In addition, another study showed that AE (30 min, 3 times a week, for 12 weeks) induced upregulation of miR-126 (an inhibitor of neovascularization) compared to baseline in HFpEF patients [43].

From the 12 included studies on CVDs, two studies reported the effects of acute exercise on miRNA expression. Xu Tianzhao reported that serum miR-21, miR-378, and miR-940 levels were upregulated immediately after acute exercise in adults with heart failure [44]. However, Barbara Mayr performed a 12 min acute maximal-cycle ergospirometry test in 20 CAD patients, illustrating that acute exercise was able to upregulate miR338-3p (a key player in the myocardial contraction pathway) and downregulate miR101-3p (anti-atherogenic) expression [41]. Another study reported that both aerobic interval training (AIT) for 15 min, 3 times a week, and moderate continuous training (MCT), 46 min of walking per week, performed for 3 months, equally downregulated miR-15a-5p, miR-93-5p, and miR-451a (the markers of plaque vulnerability) in 24 CAD patients [40]. Moreover, Tai Chi performed for 12 weeks downregulated miR-126 expression in adults affected by CAD [45].

Exercise training for 5–20 min on a bicycle ergometer for 12 weeks attenuated pro-angiogenic circulating miRs, i.e., miR-126 and miR-21, expression in chronic heart failure (CHF) patients (63 ± 3 years), while miR-221, miR-222, and miR-214 did not change [46]. Sieland analyzed miRNA expression before and after vigorous and moderate-intensity bouts of walking exercise in older adults with peripheral arterial disease with claudication. Results showed that miRNA-142-5p and miRNA-424-5p were upregulated only during the moderate-intensity intervention [47].

## 3. Discussion

The aim of this systematic review was to comprehensively evaluate the current literature to consolidate information on the relationship between physical activity/exercise and the expression of microRNAs (miRNAs) in different biological samples (i.e., plasma, serum, platelets, and muscle tissues) associated with the prevention, treatment, and survival of NCD-affected patients. The goal of this research was to review the state-of-the-art on the miRNAs’ expression mediated by different types of exercises in different NCDs, evidencing the possible role of miRNAs as prognostic markers and the association between miRNAs’ expression and beneficial marker outcomes in NCD patients. We found five articles regarding cancers, thirteen on CVDs, nine on T2DM, and two on COPD, in which the role of different types of exercises on the expression of some miRNAs was associated with targeting different molecular pathways in NCD patients.

To make the review more understandable, the findings from each NCD investigated were reviewed separately, as shown below.

### 3.1. Cancers

As a NCD, cancer poses a social, economic, and clinical burden on society, as it is the second highest cause of mortality around the globe [48]. Among all cancer types, prostate cancer (PC) is the most frequent form of cancer among men, whereas breast cancer (BC) is the most common form among women [49]. In the current systematic review, our findings underscore the multifaceted impact of exercise (acute as well as chronic) on the upregulation of some circulating miRNAs, i.e., miR-133, miR-1, miR-29b, miR-370-3p, miR-211, miR-205, miR-10a, and miR-206, and the downregulation of miR-27a, miR-21, miR-155, miR-221, and miR-10b, labeled as oncomiRs, thereby providing strong evidence of the association between exercise and oncomiR expression regulation and tracking a potential mechanism through which exercise may contribute to cancer prevention and management [21,22,23,24,25].

Dysregulation of some miRNAs has been linked to breast cancer (BC) progression, with some miRNAs serving as tumor suppressors and others showing oncogenic capabilities. Notably, miR-133a and miR-133b have been found to be important tumor suppressors, with decreased levels of miR-133a associated with increased cell migration in breast cancer tissues [50]. This review evidenced that treadmill-walking and weight training could increase miR-133a-3p levels in blood serum of BC patients, indicating a possible role in delaying cancer progression [23]. This finding is congruent with Gazova, who reported elevated circulating levels of miR-133a in BC patients performing strength training [21]. Additionally, the miR-143/145 cluster, known as a tumor-suppressing player in BC, was upregulated by exercise, predicting the effective role of exercise in tumor suppression [24].

The let-7 miRNA family, known for its role in cancer growth and glucose metabolism regulation, acts as a tumor suppressor [51]. Physical activity has been demonstrated to significantly influence the overexpression of let-7a and let-7b in BC patients who engage in both low- and high-intensity exercise [24,25]. Furthermore, in vivo studies indicate that overexpression of miR-1 targeted Bcl-2 to decrease the tumor volume and weight in nude mice [52]. Similarly, in BC patients undergoing exercise training, miR-1 was upregulated, in association with the post-transcriptional regulation of crucial tumor-suppressor genes, indicating that physical activity may play a role in tumor suppression, myogenesis, and increased muscle strength in cancer patients [21,23].

Olson and Alizadeh reported a significant upregulation of specific microRNAs (miR-211, miR-205, miR-10a, and miR-206) following bouts of exercise [22,24]. The increased expression of miR-10a-5p was associated with anti-tumor effects in breast cancer cells by inhibition of the PI3K-AKT pathway and the stathmin pathway, as previously reported by Zhang [45]. Similarly, the upregulation of miR-205-5p was identified as a favorable clinical prognostic factor in breast cancer tissues for tumor reduction [53]. Moreover, miR-206 may exert anti-angiogenic effects in breast tumors, while upregulation of miR-211-5p has been shown to decrease breast cancer cell viability and induce apoptosis, contributing to a reduction in tumor size [24]. This collective evidence underscores the intricate role of microRNAs in modulating molecular pathways and influencing breast cancer tumor reduction following exercise.

In contrast, miR-191, an estrogen-responsive miRNA, functions as an oncogenic regulator in breast cancer, promoting proliferation, migration, and therapeutic resistance. Interestingly, Adams observed an upregulation of miR-191 after exercise, which could be influenced by hormone therapy in patients, showcasing a potential contrasting effect [26].

BC recurrence is also a concern in obese women. Adams and co-workers highlighted the potential significance of aerobic exercise and weight loss intervention correlated with body mass index (BMI) in downregulation of miR-106b and miR-92a. These miRNAs target cell-cycle regulators in BC cells and serve as prognostic indicators of BC recurrence, suggesting that exercise can reduce the chances of recurrence [25].

Over-expression of oncomiRs targets the genes involved in the regulation of phosphatase and tensin homolog (PTEN), programmed cell death protein 4 (PDCD4), and signal transducer activator of transcription 3 (STAT3) pathways. These miRNAs have been associated with different hallmarks of cancer cells, including cell proliferation, cell motility, metastasis, and drug resistance [54]. Via concomitant downregulation of oncomiRs, such as miR-21, miR-155, miR-221, and miR-10b, as a consequence of exercise intervention in cancer tissues of patients, these miRNAs suggest a novel therapeutic approach for cancer treatments [24].

A previous study explained the involvement of miR-27 in the migration and invasion of breast cancer by targeting the SFRP1 gene via the Wnt/β-catenin signaling pathway [55]. Two studies reported the downregulation of miR-27a after exercise intervention, suggesting that physical activity may play a crucial role in controlling cancer invasion [24,25].

Despite these intriguing findings, further research is warranted to unravel the precise molecular pathways involved and to establish the clinical implications of the observed miRNA alterations in the context of cancer prognosis and recurrence.

### 3.2. Type 2 Diabetes Mellitus (T2DM)

Diabetes is one of the four major types of NCDs and it occurs when the body does not produce enough insulin or cannot effectively use the produced insulin [56]. T2DM individuals have a high risk of CVD [57]. Indeed, these patients show a prothrombotic state that is usually attributed to platelet dysfunction [58]. Downregulation of miR-130a resulted in increased platelet activation in T2DM [59]. Although Akbarinia showed that miR-130a expression increased after eight weeks of aerobic training, these changes were not statistically significant when compared with the control group [26]. Instead, a prospective observational study found that miR-130a expression was upregulated in the moderate exercise group monitored for 12 weeks [27]. This upregulation of miR-130a through exercise could be a significant mediator in maintaining the platelet dysfunction in T2DM. Moreover, the expression of hsa-miR-223 in plasma and platelets is a marker of thrombotic events in individuals affected by T2DM. Previously, a study on a mouse model demonstrated that miR-223 knockout impairs the recovery of platelet production following platelet immuno-depletion, highlighting the role of miR-223 in thrombopoiesis [60]. However, eight weeks of aerobic training had no effects on hsa-miR-223 expression, as reported by Taghizadeh [33], whereas an upregulation of miRNA-223 was reported after twelve weeks of aerobic training [34]. Exercise duration may effectively influence the regulation of miRs involved in platelet function and thrombotic events in individuals affected by T2DM.

MicroRNA-146a mitigates inflammation in T2DM by targeting interleukin-1 receptor-associated kinase 1 (IRAK1)/TNF receptor-associated factor 6 (TRAF6), thereby reducing inflammatory cytokine production and regulating the genes involved in the pathogenesis of T2DM [61]. Cirilli have shown that high-intensity exercise downregulated microRNA-146a [27]. Two other studies investigating miR-146a expression revealed an upregulation after an acute resistance training (RT) session [28] and after eight weeks of a combination of RT and HIIT [29]. The upregulation of miR-146a in response to exercise predicts a reduction in T2DM’s complications by influencing chronic inflammation, a key factor in insulin resistance and microvascular complications.

When analyzing skeletal muscle samples, regulation of the transcriptome downstream of miR-29a suggests a novel epigenetic mechanism controlling therapeutically functional vascular plasticity in aerobic training. This finding warrants further investigation for its potential association with therapeutic interventions for vascular disease in T2DM. Instead, RT upregulated miR-23a and miR-195 suggests an involvement of these miRNAs in blood vessel development [31]. Another study investigating skeletal muscles’ miRNA samples illustrated that miRNA-29b-3p, miRNA-29c-3p, and miRNA-135a-5p levels were downregulated after 12 weeks of aerobic training [32]. Moreover, miR-29b was downregulated following combined RT and HIIT performed on separate days [29]. Some of these miRNAs (29b-3p, 29c-3p, and 135a-5p) were found to negatively affect glucose metabolism [32]. Therefore, physical activity may represent an important tool in order to regulate glycemia due to the downregulation of the mentioned miRNAs.

Post-training changes of several c-miRNAs (namely, miR-451a, miR-423-3p, and miR-766-3p) seem to occur irrespective of exercise type (aerobic or resistance) in individuals with T2DM [31]. Change from baseline miR-451a and miR-423-3p expression appeared to be strongly associated with total fat loss. However, the most convincing association was observed for miR-451a because it was not linked exclusively to exercise types, but also to other relevant metabolic variables (age, sex, and baseline glycemic control).

### 3.3. Chronic Obstructive Pulmonary Disease (COPD)

Chronic obstructive pulmonary disease (COPD) is a common lung disease causing restricted airflow and breathing problems [62]. Muscle-specific miRNAs’ (myomiRs) expression, including miR-1, miR-206, miR-133, miR-208, and miR-499, may also be dysregulated and contribute to skeletal muscle weakness in COPD. Downregulation of miR-133 and miR-206 reduced muscular strength in respiratory muscles, while the histone deacetylase 4 (HDAC4) and myocyte enhancer factor 2 (MEF2) protein levels were increased and the exercise tolerance in COPD patients was decreased. miR-133 and miR-206 were negatively correlated with daily physical activity in COPD patients [37]. Interestingly, upregulated hsa-let-7c and downregulated hsa-miR-1277 during exercise in COPD played an essential role in our study on miRNA networks. Indeed, overexpressing hsa-miR-1277 may reduce IL-1β-induced CHON-001 cell damage and slow the course of Parkinson’s disease. A pilot study revealed that miR-144-3p and hsa-miR-1277 were downregulated after 12 weeks of an individualized AT, whereas hsa-let-7c was upregulated, elaborating on the impact of exercise in COPD patients [36]. Future mechanistic studies are needed to determine the effect of exercise on COPD and the activity of miR-144-3p and other c-miRNAs.

### 3.4. Cardiovascular Diseases (CVDs)

The worldwide prevalence of cardiovascular risk factors and mortality rate have progressively increased. Smoking, diabetes mellitus, obesity, and hypertension are identified as some of the most prevalent causes of premature death due to CVDs [60]. In this regard, the vasoprotective and anti-inflammatory microRNAs could be of great importance to reduce hypertension. The miR-146a is known as a high-shear-stress-inducible micro-RNA, and it can inhibit the NF-κB pathway, reducing endothelial inflammation, and ultimately reducing hypertension. It has been shown that 16 weeks of aerobic exercise (AE) may upregulate miR-146 in HFpEF patients [10,37], and 12 weeks of AE in CAD patients [38]. This upregulation decreased muscle inflammation in HFpEF and the severity of CVD due to the anti-inflammatory effect in CAD [37,38].

Acute exercise in CAD patients downregulated anti-atherogenic miR101-3p [43], which counteracted plaque formation. Similarly, exercise training attenuated the reduction of miR-126 induced by high-density lipoproteins (HDL) in CHF, thereby preventing atherogenesis and endothelial dysfunction [46]. Both aerobic interval training (AIT) and moderate continuous training (MCT) downregulated the miR-15a-5p, miR-93-5p, and miR-451a expression after exercise, indicating that lower levels of these miRNAs may be related to coronary atherosclerosis regression in CAD patients [38]. Another miR-142-5p expression is related to apoptosis in human macrophages by targeting TGF-β2. This effect could play an important role in the progression of atherosclerosis [63]. Moderate physical activity can also upregulate the expression of miR-142-5p and miR-424-5p [45], reducing the risk of plaque formation in older adults with peripheral arterial disease.

Another problem in CVDS patients is reduced neovascularization. Physical activity may have a positive effect in preventing vascular inflammation and retaining neovascularization. Indeed, it was observed that aerobic exercise upregulated neovascularization, inducing miR-126 expression in heart failure patients [43]. A previous study on a mouse model showed that exercise could reduce lipogenesis through the downregulation of miR-34a in hepatocytes [64]. Witvrouwen showed that 15 weeks of combined strength training and AT reduced miR-146a levels in adults affected by HFpEF [39]. In contrast, Zhang and colleagues reported a downregulation of c-miR-126 expression after 3 months of Tai Chi training in individuals with CHD and adipose tissue dysfunction, suggesting that Tai Chi practice reduces the risk of CHD through MAPK/ERK pathway regulation and indicating miR-126 as a possible marker for CHD therapy [65].

CVD patients have reduced muscle functional capacity [66]. According to Antunes-Correa, 16 weeks of AT increased miR-1 expression, decreased PTEN protein expression, and reduced inhibitory action on the PI3K-AKT pathway, resulting in increased muscle functional capacity and blood flow in HFpEF patients [40]. In contrast, the maximum-cycle ergospirometry test in CAD patients was not effective in regulating miR-1 [41], suggesting the positive effect of chronic aerobic exercise on increasing the functional capacity of muscles in CVD patients. Similarly, miR-181c, a pro-fibrotic responsive miRNA, prevented heart failure by inhibiting cardiomyocyte apoptosis via the PI3K/Akt pathway [67,68]. Individuals with low compliance to PA showed a downregulated level of miR-181c [42]. Moreover, acute exercise in CAD patients reduces the risk of heart attack by promoting the regulation of miR-338-3p, which is considered a key player in the myocardial contraction pathway [41]. In failing hearts, miR-21 levels increase selectively in fibroblasts, enhancing ERK-MAP kinase activity by inhibiting sprouty homologue 1 (Spry1). This affects fibroblast survival, growth factor secretion, interstitial fibrosis, and cardiac hypertrophy. A study involving in vivo silencing of miR-21 with an antagomir in a mouse model of pressure-overload-induced disease demonstrated reduced cardiac ERK-MAP kinase activity, interstitial fibrosis, and cardiac dysfunction [69]. In this regard, 12 weeks of aerobic exercise training downregulated miR-21, suggesting a positive effect of physical activity on controlling cardiac dysfunction in CHF patients [48].

Seven miRNAs (Let-7b, miR-23a, miR-140, miR-146a, miR-191, miR-210, and miR-339-5p) involved in angiogenesis, skeletal muscle function, and inflammation processes are upregulated after exercise [39]. Therefore, these miRNAs could act as epigenetic markers of physical fitness and exercise-induced cardiovascular adaptation, predicting a promising approach for the prescription of personalized exercise [39].

Furthermore, serum miR-21, miR-378, and miR-940 expression increased in response to an acute exhaustive exercise in individuals with CHF [44]. These dysregulated miRNAs are different from those reported in individuals not affected by CVDs, indicating a distinct exercise adaptation in CHF patients. Future studies aiming at the direct biological function of circulating miRNAs in adaptation to exercise training are highly needed in individuals affected by CVDs.

## 4. Materials and Methods

### 4.1. Information Sources 

This systematic review was conducted following the guidelines established by the Preferred Reporting Items for Systematic Reviews and the Meta-Analyses (PRISMA) statement [70]. It was registered in the International Prospective Register of Systematic Reviews—PROSPERO—with code ID CRD42023463666.

### 4.2. Search Strategy

The electronic databases selected for investigation were PubMed, Scopus (Elsevier, Amsterdam, The Netherlands), MEDLINE (Medical Literature Analysis and Retrieval System on-line), and Web of Science. The literature search was conducted on 12 June 2023. A systematic search was carried out using the following keywords: “cancer”, “cardiovascular disease”, “diabetes”, “chronic diseases”, “non-communicable diseases”, “physical exercise”, “physical activity”, “exercise training”, “aerobic exercise”, “resistance exercise”, “miRNA expression”, “miR-”, and “microRNA”. Results are reported in Table 1.

### 4.3. Eligibility Criteria

All the studies resulting from the search were reported on an electronic spreadsheet and duplicates were removed using the Mendeley software version 2.112.0. 

Studies were further analyzed and deemed eligible according to the following inclusion criteria: (a) clinical trials and observational studies, (b) a focus on miRNAs and physical activity, (c) the presence of different human characteristics (male and female, individuals with non-communicable diseases, and individuals above 18 years), (d) comparisons with another intervention or a healthy control group, (e) having been indexed in previously selected databases, and (f) published in English. The exclusion criteria were as follows: (a) review articles, (b) book chapters, (c) studies using animal and in vitro models, (d) studies not addressing the effect of physical activity on miRNA, and (e) studies based on physiotherapeutic interventions. 

### 4.4. Data Collection

Two authors (M.A and F.G) of this study independently evaluated the titles and abstracts of all the articles previously identified using the search strategy. Similarly, each of the above authors evaluated the full articles and made their selections according to the set eligibility criteria. There was no disagreement between the reviewers.

### 4.5. Data Extraction

The following data were extracted from the eligible studies and are listed in Table 2, Table 3, Table 4 and Table 5: identification of the article (first author and year of publication), studied disease, studied population (participants’ characteristics, including sex and mean age), sample type, analyzed miRNAs, type of physical activity, duration and intensity of physical activity, correlation between miRNA and physical activity, miRNA expression after exercise sessions, and its function. 

### 4.6. Risk of Bias (Quality) Assessment

The risks of bias across studies was examined separately by two reviewers through careful evaluation of information based on the aspects of the research regarding the inclusion criteria, i.e., (i) methods, (ii) results, and (iii) conclusions. With regard to randomized studies, internal validity was assessed using the Risk of Bias (RoB 2) Tool by the Cochrane Foundation (2020). Regarding the non-randomized studies, internal validity was assessed using the Newcastle-Ottawa Quality Assessment Form. The assessment carried out after the evaluation is discussed as a narrative synthesis in the Discussion Section. The quality assessment results of each study are reported in Supplementary File S1 (Figures S1–S4).

## 5. Conclusions

In this systematic review, we focused on the state-of-the-art concerning the key drivers of disease progression in some NCDs, i.e., cancer, CVD, T2DM, and COPD. Although in most cases exercise protocols for NCD patients are not completely elicited, exercise modulates the expression of tissue-specific miRNAs and c-miRNAs associated with NCD improvement. Specific exercise protocols for NCD patients and inconsistencies in reporting exercise modalities pose challenges in understanding how exercise affects microRNA (miRNA) expression. Among these, miR-146, miR-181, miR-133, miR-21, and miRNA-1 families showed the best potential as miRNAs in terms of future perspectives, serving as potential non-invasive biomarkers for diagnosis, prognosis, and therapy response prediction in many of NCDs.

## Figures and Tables

**Table 1:** Search strategy carried out on each database (PubMed, Scopus, and Web of Science) on 12 June 2023.

Databases	Search Query
PubMed	((cancer [Title/Abstract] OR cardiovascular disease [Title/Abstract] OR diabetes [Title/Abstract] OR chronic diseases [Title/Abstract] OR non-communicable diseases [Title/Abstract])) AND ((physical exercise [Title/Abstract] OR physical activity [Title/Abstract] OR exercise training [Title/Abstract] OR aerobic exercise [Title/Abstract] OR resistance exercise [Title/Abstract] AND ((miRNA expression [Title/Abstract] OR microRNA [Title/Abstract] OR miR- [Title/Abstract]))
Scopus	( TITLE-ABS-KEY ( cancer ) OR TITLE-ABS-KEY ( cardiovascular AND diseases ) OR TITLE-ABS-KEY ( diabetes ) OR TITLE-ABS-KEY ( chronic AND diseases ) OR TITLE-ABS-KEY ( non AND communicable AND diseases ) AND TITLE-ABS-KEY ( physical AND exercise ) OR TITLE-ABS-KEY ( physical AND activity ) OR TITLE-ABS-KEY ( exercise AND training ) OR TITLE-ABS-KEY ( aerobic AND exercise ) OR TITLE-ABS-KEY ( resistance AND exercise ) AND TITLE-ABS-KEY ( miRNA AND expression ) OR TITLE-ABS-KEY ( microRNA ) OR TITLE-ABS-KEY ( miR- ) ) AND ( LIMIT-TO ( DOCTYPE , “ar” ) ) AND ( LIMIT-TO ( LANGUAGE , “English” ) )
Web of Science (MEDLINE)	#4((((TS=(cancer)) OR TS=(cardiovascular disease)) OR TS=(diabetes )) OR TS=( chronic diseases)) OR TS=(non-communicable diseases) #5((((TS=(physical exercise )) OR TS=(physical activity)) OR TS=(exercise training)) OR TS=(aerobic exercise)) OR TS=(resistance exercise)#6((TS=(miRNA expression )) OR TS=(microRNA)) OR TS=( miR- )#6 AND #5 AND #4 and Journal Article (Publication Type) and English (Languages)

**Table 2:** Schematic representation of study characteristics and the effects of exercise on miRNA expression in some types of cancer.

Author and Year	Study Population (Disease, Gender, and Age)	Sample Type Used for miRNA Extraction	Type, Frequency, and Duration of Exercise	Exercise Effects on miRNA Expression
Gazova et al. (2019) [21]	Prostate Cancer, 23 patients, 60–78-year-old males, randomized in only ADT vs. trained ADT	Blood plasma	Strength training (3 times/week for 16 weeks)	↑ miR-1, miR-29b, miR-133a
Olson et al. (2021) [22]	35 females, clinically stable breast cancer patients	Blood plasma	Moderate physical exercise and strength training (150 min, 2 times/week for 12 weeks)	↑ miR-10a, miR-211, miR-205
Hagstrom and Denham (2018) [23]	Females aged 49–50 years, 15 training BC survivors vs. 9 sedentary BC	Blood serum	Resistance training on leg and chest press (60 min, 3 times/week for 16 weeks)	↑ miR-133a-3p miR-370–3p
Alizadeh et al. (2019) [24]	Breast cancer (BC), females, 15 healthy sedentary vs. 15 healthy HIIT vs. 26 BC sedentary patients in HT vs. 26 BC HIIT patients in HT	Blood serum	HIIT uphill walking (3 times/week for 12 weeks)	HIIT induces ↑ miR-206, miR-145, miR-143, miR9, let-7a ↓ miR-21, miR-155, miR-221, miR27a, miR-10b
Adams et al. (2018) [25]	Breast cancer, 100 adult female survivors in the LEAN trial, in comparison to 121 BC patients enrolled in the HOPE study	Blood serum	Diet + aerobic exercise (20 min/day for 24 weeks)	↑ miR-191, miR-24, let-7b ↓ miR-106b, miR-27a, miR-92a

ADT: androgen-deprivation therapy; LEAN: lifestyle, exercise, and nutrition; HIIT: high-intensity interval training; HOPE: hormones and physical exercise; HT: hormone therapy; ↑ upregulation; ↓ downregulation.

**Table 3:** Schematic representation of study characteristics and on the effects of exercise on miRNA expression in type 2 diabetes mellitus (T2DM).

Author and Year	Study Population (Disease, Gender, and Age)	Sample Type Used for miRNA Extraction	Type, Frequency, and Duration of Exercise	Exercise Effects on miRNA Expression
Akbarinia et al., 2018 [26]	24 females with T2DM (61.92 ± 3.63 years); AT vs. CG	Blood plasma	AT: 60–75% VO2peak; 3 times/week for 8 weeks	↑ miR-130a expression in both groups. However, there were no significant differences between AT and CG.
Cirilli et al., 2019 [27]	19 adults with T2DM (62 ± 2 years); HE, ME, and LE	Blood plasma	PA recorded for 12 weeks; HE, ME, and LE	↑ miR-130a in ME; ↓ miR-146a in HE and LE
Morais Junior et al., 2017 [28]	23 adults (13 with T2DM, 68.2 ± 5.3 years)	Blood serum	D1: Strength training (40 min) D2: walking 50 min (60–70% of heart rate reserve)	↑ miR-146a in T2D after D1
Ghodrat et al., 2022 [29]	24 females with T2DM (45–65 years); RT+HIIT (SD; *n* = 7) RT+HIIT (DD; *n* = 6) CG (*n* = 8)	Blood serum	RT at 40–70% 1-RM + low-volume HIIT for 8 weeks	↑ miR-146a in SD and DD; ↓ miR-29b in DD
Olioso et al., 2019 [30]	24 adults with T2DM (55.8 ± 7.3 years); AT (*n* = 12) RT (*n* = 12)	Blood plasma	AT: 60–65% of heart rate reserve RT: 70–80% 1RM; 60 min, 3 times/week for 24 weeks	↑ miR-423-3p, miR-451a, and miR-766-3p in AT and RT
Rowlands et al., 2014 [31]	17 adults with T2DM (49 ± 5 years); AT (*n* = 8) RT (*n* = 9)	Skeletal muscle	AT: 40–60 min of cycle ergometer RT: 2–3 sets for 6–8 repetitions; 3 times/week for 16 weeks	↓ miR-29a in AT; ↑ miR-23a and miR-195 in RT
Simaitis et al., 2020 [32]	7 adults with T2DM (61 ± 10 years)	Skeletal muscle	AT: 70–80% of peak heart rate; 3 times/week for 12 weeks	↓ miRNA-29b-3p ↓ miRNA-29c-3p ↓ miRNA-135a-5p
Taghizadeh et al., 2018 [33]	20 females with T2DM (62.3 ± 4.0 years); AT (*n* = 10) CG (*n* = 10)	Blood plasma	AT: 60–75% of VO2peak; 3 times/week for 8 weeks	hsa-miR-223 expression was not significant between AT and CG
Taghizadeh et al., 2020 [34]	24 adults with T2DM (60.0 ± 2.8 years); AT (*n* = 12) CG (*n* = 12)	Blood plasma	AT: 65–75% of VO2peak; 12 weeks	↑ miRNA-223 in AT

T2DM, type 2 diabetes mellitus; AT, aerobic training; CG, control group; HE, high-intensity exercise; ME, medium-intensity exercise; LE, low-intensity exercise; PA, physical activity; RT + HIIT (SD), resistance training + high-intensity interval training on the same day; RT + HIIT (DD), resistance training + high-intensity interval training on different days; 1-RM, one repetition maximum; D1: day 1; D2, day 2; ↑ upregulation; ↓ downregulation.

**Table 4:** Schematic representation of study characteristics and of the effects of exercise on miRNA expression in chronic obstructive pulmonary disease (COPD).

Author and Year	Study Population (Disease, Gender, and Age)	Sample Type Used for miRNA Extraction	Type, Frequency, and Duration of Exercise	Exercise Effects on miRNA Expression
Lewis et al., 2012 [35]	45 adults (31 with COPD, 65 ± 7 years)	Skeletal muscle	Daily PA	miR-133 and miR-206 expression negatively correlates with daily PA
Liu et al., 2023 [36]	4 males with COPD (60–67 years)	Blood plasma	12 weeks of individualized AT	↓ miR-144-3p ↑ hsa-let-7c ↓ hsa-miR-1277

COPD, chronic obstructive pulmonary disease; AT, aerobic training; PA, physical activity; ↑ upregulation; ↓ downregulation.

**Table 5:** Schematic representation of study characteristics and the effects of exercise on miRNA expression in cardiovascular diseases (CVDs).

Author and Year	Study Population (Disease, Gender, and Age)	Sample Type Used for miRNA Extraction	Type, Frequency, and Duration of Exercise	Exercise Effects on miRNA Expression
Witvrouwen et al. (2021) [10]	18 Male HFrEF patients (50.7–65.4 years); 9 ER vs. 9 ENR	Blood serum	Aerobic exercise 3 times, 50 min/week for 4 months	↑ Let-7b, miR-23a, miR-140, miR-146a, miR-191, miR-210, and miR-339-5p highly correlated with VO2 peak trainability in HFrEF
Taraldsen et al. (2022) [38]	24 CAD patients, aerobic interval training (AIT) vs. moderate continuous training (MCT), compared to healthy individuals (CG)	Blood serum	AIT, 15 min, 3 times/week for 3 months; MCT, 46 min walk/week for 3 months	↑ miR-146a-5p ↓ miR-15a-5p, miR-93-5p, and miR-451a
Witvrouwen et al., 2021b [39]	25 males with HFrEF (55.6 ± 13.4 years) vs. 21 CG (60.0 ± 9.4 years)	Blood plasma	3 times per week of combined strength and AT (at 90% of HR) for 15 weeks	↓ miR-146a in HFrEF patients
Antunes-Correa et al. (2020) [40]	33 (M + F) HFpEF patients (35–70 years); 11 IMT + 12 AET vs. 10 untrained CG	Muscle biopsies	Aerobic exercise (30 min, 5 times/week for 4 months)	↑ miRNA-1 ↓ PI3K-AKT
Mayr et al. (2021) [41]	20 (M + F) CAD patients (53–62 years)	Blood plasma	Maximum-cycle ergospirometry (12.44 ± 3.23 min)	↑ miR338-3p ↓ miR101-3p no change in miR-1, miR-133, miR-208a, or miR-499 expression
Gevaert et al. (2021) [42]	51 (M + F) HFpEF patients (70 ± 6 years); High responders (n = 30) Low responders (n = 21) vs. CG	Blood plasma	MCT: 35–50% of HRR, 200 min/week; HIIT: 20–50% of HRR, in 4 intervals of 200 min/week for 3 months	↓ MiR-181c in low responders compared to high responders
Jin et al. (2021) [43]	60 (M + F) HFpEF patients (55–70 years) exercise training vs. 30 healthy controls	Blood plasma	Aerobic exercise (30 min, 3 times/week for 3 months)	↑ miR-126 in HFpEF patients with exercise, as compared to baseline
Xu et al., 2016 [44]	28 males with HF (59.1 ± 1.8 years)	Blood serum	Incremental maximal cardiopulmonary exercise test	↑ miR-21 ↑ miR-378 ↑ miR-940
Zhang et al., 2020 [45]	30 adults with CHD (61.0 ± 8.1 years); TG (n = 18) CG (n = 12)	Blood serum	Tai Chi training for 12 weeks	↓ miR-126 in TG
Riedel et al. (2020) [46]	8 (M + F) CHF patients (63 ± 3 years); ET vs. 8 healthy controls doing the same exercise	Blood serum	Aerobic exercise patients: 3–6 times daily for 5–20 min on a bicycle ergometer Healthy CG: 4 × 30 min/day, 5 times/week	↓ miR-126, miR-21, and miR-222 No changes in miR-221 and miR-214 were observed
Sieland et al., 2023 [47]	10 adults with PAD (72.0 ± 7.0 years)	Blood plasma	D1: incremental walking exercise until volitional exhaustion D2: 20 min of interval training	↑ miRNA142-5p ↑ miRNA-424-5p in D2

HFrEF, heart failure with reduced ejection fraction;; IMT, inspiratory muscle training; AET, aerobic exercise training; CG, control group; CHD, coronary heart disease; CHF, coronary heart failure; CAD, coronary artery disease; AT, aerobic training; HR, heart rate; HRR, heart rate reserve; HF, heart failure; TG, Tai Chi group; PAD, peripheral arterial disease; ET, exercise training; ER, exercise responders; ENR, exercise non-responders; MCT, moderate continuous training; HIIT, high-intensity interval training; D1, day 1; D2, day 2; ↑ upregulation; ↓ downregulation.

## Supplementary Materials

The supporting information can be downloaded at: https://www.mdpi.com/article/10.3390/ijms25136813/s1.
