# Supplementary material for: The Effect of Physical Activity/Exercise on miRNA Expression and Function in Non-Communicable Diseases—A Systematic Review
Source: Int J Mol Sci. 2024 Jun 21;25(13):6813. doi: 10.3390/ijms25136813 (PMC11240922; doi:10.3390/ijms25136813)
Supplement: Supplementary file 1 [file ijms-25-06813-s001.zip › Supplementary file S1.pdf]

COHORT STUDIES

Note: A study can be awarded a maximum of one star for each numbered item within the Selection andOutcome categories. A maximum of two stars can be given for Comparability

Lewis et al. 2012

Selection

1) Representativeness of the exposed cohort
a) truly representative of the average patients with chronic obstructive pulmonary disease (COPD) in the community ❋
b) somewhat representative of the average severity of COPD in the community ❋
c) selected group of users eg nurses, volunteers
d) no description of the derivation of the cohort

2) Selection of the non exposed cohort
a) drawn from the same community as the exposed cohort
b) drawn from a different source
c) no description of the derivation of the non exposed cohort

3) Ascertainment of exposure
a) secure record (eg surgical records)
b) structured interview
c) written self report d) no description

4) Demonstration that outcome of interest was not present at start of study
a) yes
b) no

Comparability

1) Comparability of cohorts on the basis of the design or analysis
a) study controls for age-matched controls❋
b) study controls for any additional factor ❋ (This criteria could be modified to indicate specificcontrol for a second important factor.)

Outcome

1) Assessment of outcome
a) independent blind assessment ❋
b) record linkage ❋
c) self report
d) no description

2) Was follow-up long enough for outcomes to occur
a) yes (select an adequate follow up period for outcome of interest) ❋
b) no

3) Adequacy of follow up of cohorts
a) complete follow up - all subjects accounted for ❋
b) subjects lost to follow up unlikely to introduce bias - small number lost - >_______________% (select anadequate %) follow up, or description provided of those lost) ❋
c) follow up rate <______________% (select an adequate %) and no description of those lost
d) no statement

Cirilli et al. 2019

Selection

1) Representativeness of the exposed cohort
a) truly representative of the average patients with chronic obstructive pulmonary disease (COPD) in the community ❋
b) somewhat representative of the average severity of COPD in the community (marked ✗)
c) selected group of users eg nurses, volunteers
d) no description of the derivation of the cohort

2) Selection of the non exposed cohort
a) drawn from the same community as the exposed cohort (marked ✗)
b) drawn from a different source
c) no description of the derivation of the non exposed cohort

3) Ascertainment of exposure
a) secure record (eg surgical records) (marked ✗)
b) structured interview ❋
c) written self report
d) no description

4) Demonstration that outcome of interest was not present at start of study
a) yes ❋
b) no (marked ✗)

Comparability

1) Comparability of cohorts on the basis of the design or analysis
a) study controls for pre and post exercise VO2 Peak (select the most important factor) (marked ✗)
b) study controls for any additional factor ❋ (This criteria could be modified to indicate specificcontrol for a second important factor.)

Outcome

1) Assessment of outcome
a) independent blind assessment ❋
b) record linkage (marked ✗)
c) self report
d) no description

2) Was follow-up long enough for outcomes to occur
a) yes (12 weeks) (marked ✗)
b) no

3) Adequacy of follow up of cohorts
a) complete follow up - all subjects accounted for ❋
b) subjects lost to follow up unlikely to introduce bias - small number lost - >_______________% (select an

adequate %) follow up, or description provided of those lost) ❋

c) follow up rate <______________% (select an adequate %) and no description of those lost

d) no statement

Quality Assessment by ROB2

Figure S1. Cancer studies

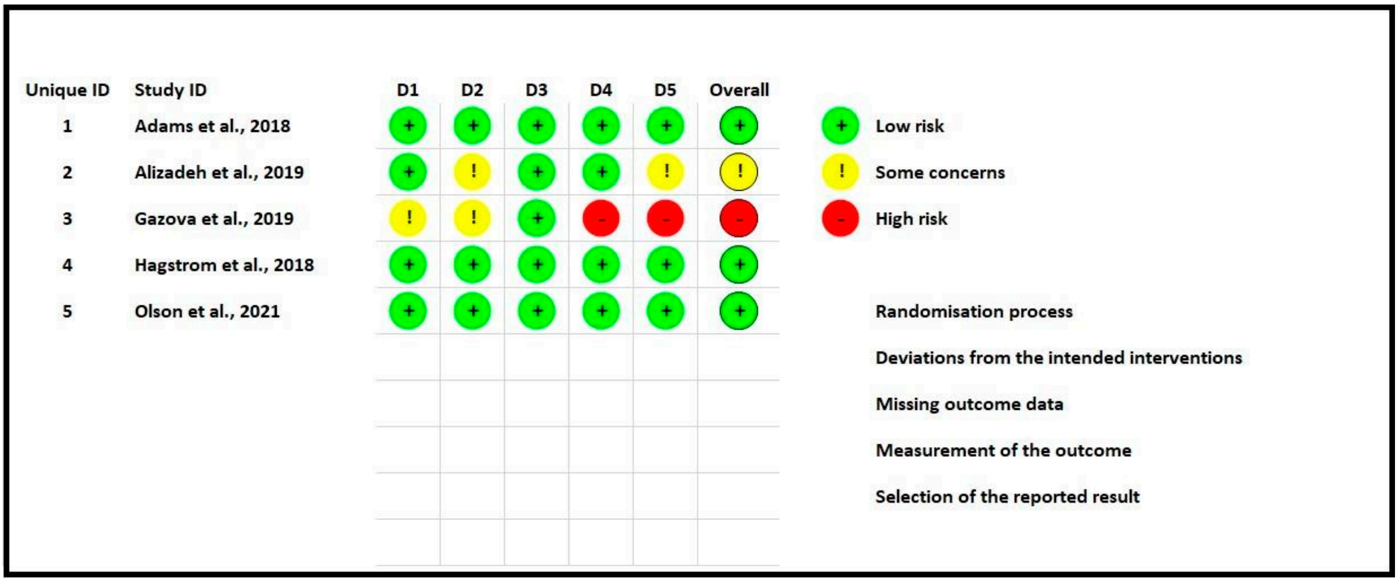

Figure S2. Type 2 diabetes mellitus (T2DM) studies

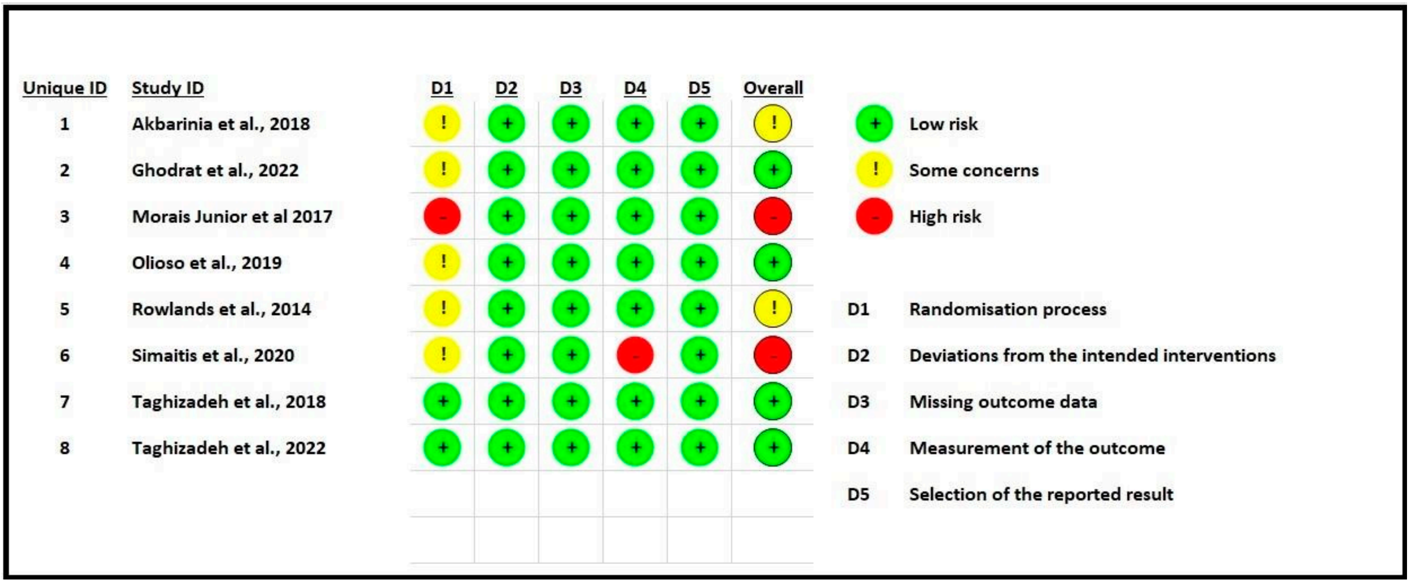

Figure S3. Chronic obstructive pulmonary disease (COPD) studies

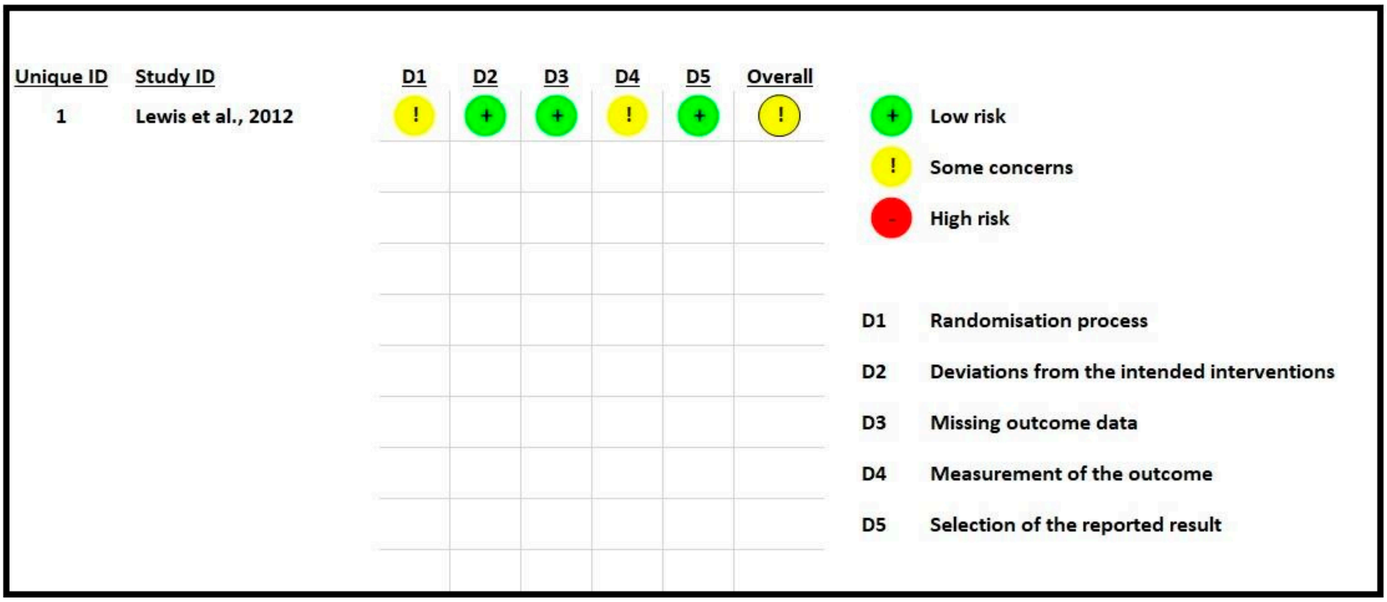

Figure S4. Cardiovascular Diseases (CVDs) studies

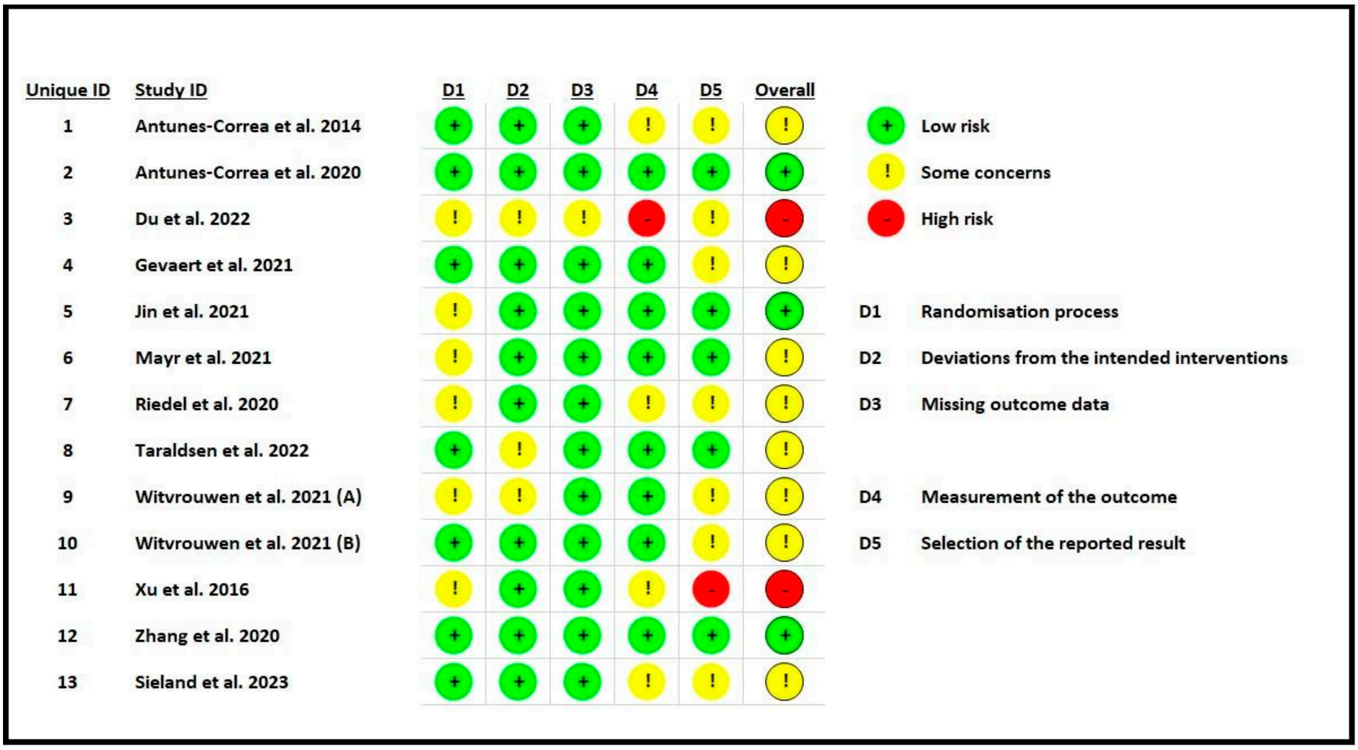

PRISMA Statement and Registration Code

This systematic review was conducted following the guidelines of Preferred Reporting Items for Systematic Reviews and Meta-Analyses PRISMA statement and registered in International Prospective Register of Systematic Reviews—PROSPERO with code ID (CRD42023463666).
